# Depressive symptoms of people living in areas with high exposure to environmental noise: a multilevel analysis

**DOI:** 10.1038/s41598-024-65497-0

**Published:** 2024-06-24

**Authors:** Il Yun, Seung Hwan Lee, Sohee Park, Suk-Yong Jang, Sung-In Jang

**Affiliations:** 1 Department of Public Health, Graduate School, Yonsei University, Seoul, Republic of Korea; 2 Institute of Health Services Research, Yonsei University, 50-1 Yonsei-to, Seodaemun-gu, Seoul, 03722 Republic of Korea; 3 Korea Health Industry Development Institute, Cheongju-si, Chungcheongbuk-do Republic of Korea; 4 Department of Biostatistics, Graduate School of Public Health, Yonsei University, Seoul, Republic of Korea; 5 Department of Healthcare Management, Graduate School of Public Health, Yonsei University, Seoul, Republic of Korea; 6 Department of Preventive Medicine, Yonsei University College of Medicine, Seoul, Republic of Korea

**Keywords:** Environmental noise, Noise pollution, Depressive symptoms, PHQ-9, Multilevel model, Health care, Signs and symptoms

## Abstract

Exposure and damage caused by noise have been reported in many countries around the world. However, few nationwide studies explored the association of residential environmental noise with depressive symptoms, this study aims to examine this association. The Korean Community Health Survey at the individual-level and the Korean Environmental Noise Measurement Database at the regional-level were used. A total of 30,630 individuals were eligible for the analysis. Multilevel model framework was applied to account for the clustered structure of the regional-level data in which individual-level data containing demographic characteristics and health information were nested. As a result of the analysis, Individuals living in the highest environmental noise area had a 1.55 times higher likelihood of experiencing depressive symptoms than those living in the lowest environmental noise area (95% CI, 1.04–2.31). After stratified analysis according to depressive symptom severity, individuals residing in areas with the highest environmental noise exposure had significantly higher odds of mild (aOR, 1.46; 95% CI, 1.02–2.07) and moderate symptoms (aOR, 1.70; 95% CI, 1.00–2.91). In conclusion, the higher the residential environmental noise, the higher the possibility of mild-to-moderate depressive symptoms. Our findings suggest the need for continued attention to and management of noise pollution, which has the potential to adversely affect individual’s mental health.

## Introduction

The World Health Organization (WHO) officially declared noise a major pollutant in 1972^1^, and for half a century it has ranked as the second most impactful environmental stressor, with a considerable proportion of urban residents experiencing chronic noise^2^. Noise greatly contributes to the degradation of living conditions and the overall quality of life in urban areas. It affects various aspects of people’s lives, including work and leisure activities, and extends beyond the industrial settings^3^. Noise stress is associated with an increased risk of disease development^4^. Numerous epidemiological studies have confirmed the long-term effects of noise pollution on metabolic, cardiovascular, and respiratory diseases^5^. A Western European study estimated that the negative effects of noise result in at least one million disability-adjusted life years (DALY) lost annually^6^. In addition, exposure to noise has been demonstrated have adverse psychological and mental effects, such as causing anxiety and depression^7–9^.

Noise is generally defined as any auditory disturbance or amalgamation of sounds that is unwanted or bothersome, causing negative impact on speech clarity, sleep quality, and other work performance^10^. Noise exposure and damage have been reported in several countries around the world. According to the US Environmental Protection Agency, in 1981 approximately 100 million people, accounting for approximately 50% of the US population, are exposed to harmful levels of traffic noise each year. In 2012, New York City received over 40,000 noise complaints^11^. The European Environment Agency has estimated that approximately 100 million people in EU member states are exposed to road traffic noise exceeding 55 dB, with 16 million exposed to railway noise, 4 million to aviation noise, and 1 million to industrial noise^12^. Similarly, in Korea, civil complaints related to noise and vibrations have increased continuously over the past decade. In 2021, 185,204 cases and approximately half of the 350,478 total environmental complaints were noise-related^13^.

Due to concerns about the potential negative health effects caused by noise exposure, national and regional studies are being actively conducted in the United States and Europe. In Korea, studies exploring the impact of aviation and occupational noise on individual health outcomes have been conducted^14–17^, and most of these evaluated the effects of noise exposure on physical health. However, no nationwide study has examined the association between environmental noise exposure and mental health. Therefore, this study aimed to identify the effects of environmental noise exposure in residential areas on depressive symptoms, using a multilevel analysis.

## Materials and methods

### Data and study population

This study used two publicly accessible data sources: first, the 2019 Korean Community Health Survey (CHS) by the Korea Centers for Disease Control and Prevention (KCDC), individual-level data, which has been collected annually from all local public health centers since 2008, was used to identify the health status of local residents for the establishment and evaluation of local health policies^18^. Second, the 2019 Korean environmental noise measurement data, region-level data, collected by the Korean Ministry of Environment for the purpose of being used as a basis for national noise management and effective noise reduction policy establishment. Automatic environmental noise monitoring devices were installed at 67 locations in 15 cities across the country, specifically targeting areas that may experience noise-related issues^13^.

Among the participants in the 2019 CHS (n = 229,099), only residents of areas where an automatic environmental noise monitoring device was installed were included (n = 34,519); those with missing values on any of the independent variables were excluded (n = 3889). A total of 30,630 individuals were included in the study. The study protocol was approved by the Institutional Review Board of the Yonsei University Health System (IRB Number: 4–2023-0364). Because the data did not contain any identifiable information, the requirement for informed consent was waived.

### Measures

The dependent variable was depressive symptoms measured using the Korean version of the Patients Health Questionnaire-9 (PHQ-9). The Korean version of PHQ-9 demonstrates good sensitivity and specificity, and setting the cutoff score at 5 points resulted in a reliable and valid tool for screening patients with depression in the general population^19,20^. In this study, the Cronbach’s α coefficient of the PHQ-9 was 0.81. If the total score of the PHQ-9 respondents was less than 5 points, they were defined as normal group, and those who scored more than 5 points were defined as the depressive symptom group. A total score of 5 or more and less than 10 was classified as the mild symptom group, 10 or more points and less than 20 points as the moderate symptom group, and 20 or more points as the severe symptom group^21^.

Environmental noise, the variable of interest, was defined as noise from all sources except the workplace^22^, that is, noise that surrounds us in our daily lives. For example, there are typically low-level sounds of leaves rustling in the wind or traffic noise heard in the distance. The noise level was expressed as the Equivalent Continuous Sound Pressure Level (Leq), which is a measure used to quantify the average sound pressure level over a given period. It represents the overall loudness or energy content of a sound considering both its intensity and duration of the sound^23^. Leq is commonly used in various fields, such as environmental noise assessments, industrial noise control, and occupational health and safety regulations^24–26^. The Korean Ministry of Environment installed a total of 67 automatic environmental noise measurement devices in 38 counties across 15 cities across the country with the purpose of measuring all daily noise. The devices are installed approximately 1 m from the road edge, and the microphone height is 4 m above the ground. In addition, since these are automatic devices, a continuous measurement mothed was applied with a sample period of less than 1 s. If there were several measurement values in one county, the average value was calculated and analyzed. The 38 county-level environmental noise measurements were divided to four quartiles (Q1: Leq ≤ 66.08 dB; Q2: 66.08 dB < Leq ≤ 68.75 dB; Q3: 68.75 dB < Leq ≤ 70.53 dB; Q4: 70.53 dB < Leq) and analyzed. In other words, from Q1 to Q4, it indicates areas with more severe exposure to environmental noise.

Socioeconomic factors (sex, age, household income, educational level, occupation, and housing type), health behavior patterns (drinking and smoking), and health condition factors (chronic diseases and subjective health status) were included. Additionally, regional-level covariates included residential area and fiscal self-reliance ratio, a representative indicator of regional financial soundness. The fiscal self-reliance ratio is the ratio of local revenue (local tax and non-tax income) to the budget of the local autonomous body^27^.

### Statistical analysis

We conducted a chi-square test to examine and compare the general characteristics of the study population. Subsequently, a multilevel model (MLM) framework was applied to account for the clustered structure of the regional-level data, in which individual-level data containing demographic characteristics and health information were nested. To estimate the parameters of MLM, we used Restricted Maximum Likelihood (REML), which leads to less biased estimates of the fixed effects and more accurate estimates of the variance components of the random effects^28,29^. Then, a multilevel logistic regression analysis was performed to explore the effect of environmental noise exposure on individual depressive symptoms in residential areas. In addition, a multinomial logistic regression model was used for stratified analysis according to the severity of depressive symptoms.

Adjusted odds ratios (aOR) and 95% confidence intervals (CI) were presented as key results. To determine whether the research model is suitable for multilevel analysis, Intraclass Correlation Coefficient (ICC) was calculated and presented^30^. The ICC indicates the amount of variance explained by regional differences in the total variance of the dependent variable. In addition, two concepts are presented together in the results of the MLM: Between-area variance refers to the variation or differences between the higher-level units (such as regions) in a hierarchical dataset. Percentage change in variation indicates the proportion of the total variance that is explained in higher-level units. For all analyses, SAS version 9.4 (SAS Institute Inc.; Cary, NC, USA) was used, and a p-value of less than 0.05 was considered statistically significant.

## Results

Table 1 shows the general characteristics of the study population. Of the 30,630 individuals eligible for the analysis, 4839 (15.8%) had depressive symptoms with a PHQ-9 score of 5 or higher. Differences in the prevalence of depressive symptoms were confirmed based on the individual characteristics. A relatively higher prevalence of depressive symptoms was reported in women over 60 years of age with low education and income, smoking, non-drinking, chronic disease, and poor subjective health status. Additionally, a high percentage of people living in areas with severe environmental noise and in small cities or rural areas suffer from depressive symptoms.

**Table 1:** General characteristics of the study population.

Variables	Depressive symptoms						
	Total		Yes		No		*P-value*
	N	%	N	%	N	%	
	30,630	100.0	4839	15.8	25,791	84.2	
Individual-level							
Sex							< .0001
Men	13,512	44.1	1561	11.6	11,951	88.4	
Women	17,118	55.9	3278	19.1	13,840	80.9	
Age							< .0001
≤ 19	379	1.2	61	16.1	318	83.9	
20 ~ 39	7903	25.8	1372	17.4	6531	82.6	
40 ~ 59	11,187	36.5	1387	12.4	9800	87.6	
60 +	11,161	36.4	2019	18.1	9142	81.9	
Education level							< .0001
Low	4242	13.8	1080	25.5	3162	74.5	
Middle	12,077	39.4	1951	16.2	10,126	83.8	
High	14,312	46.7	1809	12.6	12,503	87.4	
Occupation							< .0001
White-collar	7109	23.2	831	11.7	6278	88.3	
Blue-collar	5794	18.9	772	13.3	5022	86.7	
Pink-collar	4385	14.3	616	14.0	3769	86.0	
Else	13,342	43.6	2620	19.6	10,722	80.4	
Household Income level^a^							< .0001
Q1	9518	31.1	2092	22.0	7426	78.0	
Q2	6335	20.7	955	15.1	5380	84.9	
Q3	7798	25.5	1004	12.9	6794	87.1	
Q4	6979	22.8	788	11.3	6191	88.7	
Housing type							< .0001
Common house	13,827	45.1	2396	17.3	11,431	82.7	
Apartment	16,803	54.9	2443	14.5	14,360	85.5	
Smoking							0.0004
Yes	4953	16.2	866	17.5	4087	82.5	
No	25,677	83.8	3,973	15.5	21,704	84.5	
Drinking							< .0001
Yes	21,166	69.1	3079	14.5	18,087	85.5	
No	9464	30.9	1760	18.6	7704	81.4	
Chronic diseases							< .0001
Yes	8707	28.4	1658	19.0	7049	81.0	
No	21,923	71.6	3181	14.5	18,742	85.5	
Subjective health status							< .0001
Good	11,494	37.5	754	6.6	10,740	93.4	
Bad	19,136	62.5	4,085	21.3	15,051	78.7	
Region-level							
Environmental noise^b^							< .0001
Q1	5742	18.7	687	12.0	5055	88.0	
Q2	8596	28.1	1560	18.1	7036	81.9	
Q3	5612	18.3	869	15.5	4743	84.5	
Q4	10,680	34.9	1723	16.1	8957	83.9	
Residential area							< .0001
Seoul	5661	18.5	930	16.4	4731	83.6	
Metropolitan cities	17,128	55.9	2375	13.9	14,753	86.1	
Small cities and rural	7841	25.6	1534	19.6	6307	80.4	
Fiscal self-reliance ratio^c^							< .0001
Q1	7948	25.9	1412	17.8	6536	82.2	
Q2	10,020	32.7	1201	12.0	8819	88.0	
Q3	7001	22.9	1296	18.5	5705	81.5	
Q4	5661	18.5	930	16.4	4731	83.6	

^a^Household income level was categorized into quartiles (Q1: monthly income ≤ 2 million won ($1,500); Q2: 2 million won ($1,500) < monthly income ≤ 3.5 million won ($2,600); Q3: 3.5 million won ($2,600) < monthly income ≤ 5 million won ($3,700); Q4: 5 million won($3,700) < monthly income).

^b^Environmental noise measurements were divided to quartiles (Q1: Leq ≤ 66.08 dB; Q2: 66.08 dB < Leq ≤ 68.75 dB; Q3: 68.75 dB < Leq ≤ 70.53 dB; Q4: 70.53 dB < Leq).

^c^Fiscal self-reliance ratio were divided to quartiles (Q1: ratio ≤ 40.8%; Q2: 40.8% < ratio ≤ 50.0%; Q3: 50.0% < ratio ≤ 60.5%; Q4: 60.5% < ratio).

Table 2 presents the results of the multilevel analysis of the association between regional environmental noise and individual depressive symptoms. In this framework, the ICC value of the null model was 5.9%, confirming a clustering effect^31^. Regional environmental noise exposure had a significant effect on individual depressive symptoms in the full model (Model 3) after adjusting for all covariates. When the level of environmental noise was classified into four quartiles, the more severe the environmental noise in the residential area, the higher the likelihood of depressive symptoms, increasing linearly. The possibility of developing depressive symptoms was 1.55 times higher for individuals living in the highest environmental noise area [Q4] compared to individuals living in the lowest level of environmental noise [Q1] (95% CI, 1.04–2.31).

**Table 2:** Adjusted odds ratios of depressive symptoms by characteristics of individual- and region-level (multilevel model).

Variables	Depressive symptoms					
	Model 1		Model 2		Model 3	
	aOR	95% CI	aOR	95% CI	aOR	95% CI
Individual-level						
Sex						
Men	1.000				1.000	
Women	1.767	(1.631–1.915)			1.766	(1.630–1.914)
Age						
≤ 19	1.000				1.000	
20 ~ 39	1.094	(0.811–1.477)			1.099	(0.814–1.484)
40 ~ 59	0.546	(0.404–0.738)			0.549	(0.406–0.742)
60 +	0.464	(0.341–0.631)			0.466	(0.342–0.634)
Education level						
Low	1.715	(1.511–1.947)			1.715	(1.511–1.946)
Middle	1.263	(1.155–1.382)			1.262	(1.153–1.380)
High	1.000				1.000	
Occupation						
White-collar	1.000				1.000	
Blue-collar	0.936	(0.829–1.056)			0.935	(0.828–1.055)
Pink-collar	0.996	(0.881–1.125)			0.996	(0.881–1.125)
Else	1.267	(1.144–1.404)			1.267	(1.144–1.403)
Household Income level^a^						
Q1	1.750	(1.571–1.951)			1.758	(1.577–1.959)
Q2	1.264	(1.132–1.411)			1.266	(1.134–1.413)
Q3	1.159	(1.043–1.288)			1.159	(1.043–1.288)
Q4	1.000				1.000	
Housing type						
Common house	1.062	(0.990–1.138)			1.060	(0.989–1.137)
Apartment	1.000				1.000	
Smoking						
Yes	1.668	(1.511–1.841)			1.668	(1.511–1.841)
No	1.000				1.000	
Drinking						
Yes	0.929	(0.861–1.002)			0.928	(0.860–1.001)
No	1.000				1.000	
Chronic diseases						
Yes	1.135	(1.046–1.232)			1.134	(1.045–1.231)
No	1.000				1.000	
Subjective health status						
Good	1.000				1.000	
Bad	3.444	(3.160–3.754)			3.445	(3.160–3.755)
Region-level						
Environmental noise^b^						
Q1			1.000		1.000	
Q2			1.010	(0.514–1.987)	1.247	(0.682–2.280)
Q3			1.360	(0.879–2.104)	1.355	(0.922–1.992)
Q4			1.419	(0.907–2.218)	1.547	(1.035–2.312)
Residential area						
Seoul			1.000		1.000	
Metropolitan cities			1.624	(0.773–3.411)	1.393	(0.685–2.831)
Small cities and rural			1.870	(0.815–4.291)	1.703	(0.770–3.765)
Fiscal self-reliance ratio^c^						
Q1			1.417	(0.886–2.267)	1.282	(0.815–2.018)
Q2			0.811	(0.528–1.244)	0.747	(0.494–1.129)
Q3			1.648	(0.782–3.474)	1.431	(0.697–2.939)
Q4			1.000		1.000	
Between area variance (SE)	0.2038 (0.04990)*		0.1312 (0.03278)*		0.1182 (0.02981)*	
Percentage change in variation	0.5%		35.9%		42.3%	
Model fitness						
-2 Log Likelihood	23,771.98		25,962.940		23,902.100	
Intraclass correlation coefficient (%)	5.8%		3.8%		3.5%	

Model 1: Adjusting only individual-level independent variables; Model 2: Adjusting only region-level independent variables; Model 3: Adjusting all independent variables (full model).

Intraclass correlation coefficient of null model: 5.9%.

*aOR* adjusted odds ratio, *CI* confidence interval, *SE* standard error.

*p < .0001.

^a^Household income level was categorized into quartiles (Q1: monthly income ≤ 2 million won ($1,500); Q2: 2 million won ($1,500) < monthly income ≤ 3.5 million won ($2,600); Q3: 3.5 million won ($2,600) < monthly income ≤ 5 million won ($3,700); Q4: 5 million won($3,700) < monthly income).

^b^Environmental noise measurements were divided to quartiles (Q1: Leq ≤ 66.08 dB; Q2: 66.08 dB < Leq ≤ 68.75 dB; Q3: 68.75 dB < Leq ≤ 70.53 dB; Q4: 70.53 dB < Leq).

^c^Fiscal self-reliance ratio were divided to quartiles (Q1: ratio ≤ 40.8%; Q2: 40.8% < ratio ≤ 50.0%; Q3: 50.0% < ratio ≤ 60.5%; Q4: 60.5% < ratio).

Table 3 presents the results of the stratified analysis according to the severity of depressive symptoms. People living in areas with the highest exposure to environmental noise [Q4] were 1.46 times (95% CI, 1.02–2.07) and 1.70 times (95% CI, 1.00–2.91) more likely to develop mild and moderate depressive symptoms, respectively. In other words, severe environmental noise in residential areas increases the risk of mild-to-moderate depressive symptoms. In addition, subgroup analysis was performed according to all independent variables included in the analysis, and the results are shown in the Supplementary Table.

**Table 3:** Results of subgroup analysis stratified by severity of depressive symptoms.

Variables	Depressive symptoms						
	Normal (PHQ-9 < 5)	Mild (5 ≤ PHQ-9 < 10)		Moderate (10 ≤ PHQ-9 < 20)		Severe (PHQ-9 ≥ 20)	
	aOR	aOR	95% CI	aOR	95% CI	aOR	95% CI
Environmental noise^a^							
Q1	1.00						
Q2		1.19	(0.70–2.01)	1.43	(0.65–3.15)	1.01	(0.22–4.51)
Q3		1.24	(0.89–1.74)	1.60	(0.96–2.67)	1.26	(0.52–3.06)
Q4		1.46	(1.02–2.07)	1.70	(1.00–2.91)	1.17	(0.46–3.02)

*aOR* adjusted odds ratio, *CI* confidence interval.

^a^Environmental noise measurements were divided to quartiles (Q1: Leq ≤ 66.08 dB; Q2: 66.08 dB < Leq ≤ 68.75 dB; Q3: 68.75 dB < Leq ≤ 70.53 dB; Q4: 70.53 dB < Leq).

## Discussion

This study examined the effects of environmental noise exposure on depressive symptoms in residential areas using a MLM. The main findings of this study are as follows: first, as environmental noise exposure in the residential area increased, the odds of depressive symptoms tended to increase, and the likelihood of developing depressive symptoms was 1.55 times higher in residents in areas with the most severe environmental noise than in those with the least environmental noise. Second, the likelihood of mild-to-moderate symptoms was significantly higher in those living in areas with the highest exposure to environmental noise. In other words, heightened levels of environmental noise in residential areas increase the risk of developing mild-to-moderate depressive symptoms. Meanwhile, exposure to environmental noise did not affect the severe depressive symptoms, which is predicted to be a result of the fact that environmental noise is like a constant background sound that surrounds our daily lives, so it can be a factor that harms mental health to some extent, but there is no risk of causing serious depression.

Our findings support previous studies that have identified the negative impact of noise on health and quality of life. It has been reported that continuous exposure to noise not only causes sleep and communication disturbance^32,33^. In particular, similar to this study, there was an investigation that examined the impact of regional traffic noise on depressive symptoms. In their findings, people exposed to significant traffic noise had a 1.29 times higher relative risk of depressive symptoms than those exposed to lower levels of noise^34^. In addition, our results align with those of previous study, which confirmed the association between workplace noise exposure and mild depressive symptoms in workers using the PHQ-9 index^35^. Drawing from both prior research and our own findings, it has come to light that exposure to noise has adverse psychological impacts. This suggests that the need for improvements, as the decline in mental well-being and health-related quality of life caused by noise can impose a social burden. Especially in Korea, where urban and metropolitan areas face significant social challenges stemming from population density, it is crucial to develop localized environmental strategies to address noise pollution.

Noise pollution, which can be harmful to health, is garnering increasing attention worldwide, and various evidence-based regulations are being implemented; however, few domestic studies have evaluated the effects of regional noise on individual health. To the best of our knowledge, this study is the first to examine the association between regional environmental noise exposure and individual depressive symptoms using data combining regional environmental noise measurement data and CHS containing individual health information. This study is different from prior studies in that the analysis was performed by applying a MLM considering the clustering effects of two highly representative datasets at the individual and regional levels. In addition, external validity is high when a large sample is included. The present study is meaningful because it provides an appropriate basis and evidence for establishing future health policies related to noise regulation and management.

However, this study had a few limitations. First, the CHS data used in this study were secondary data, and it was not possible to conduct a time-series analysis to track changes in individual health status because the survey items differed annually. Second, among the CHS participants, only residents of areas with automatic environmental noise measurement networks were selected as study samples, and there was a limitation in that only 38 (14.9%) of the 255 regions could be analyzed. In addition, the measured regional environmental noise areas were predominantly locations prone to noise problems or heavy traffic, making it difficult to compare and analyze the calm areas and areas with little traffic. Third, individual depressive symptoms were measured using the PHQ-9, a self-report questionnaire. Therefore, there was a concern that the results may vary depending on the degree of understanding and honesty of the examinee. Fourth, we attempted to analyze inter-floor noise, which has recently attracted attention as a social problem, but could not be analyzed because of the absence of certified measurements related to inter-floor noise. Finally, although we attempted to adjust for various covariates that may have affected the outcome variable, residual confounders from the unmeasured variables could not be ruled out.

## Conclusion

This study demonstrated that living in areas with high exposure to environmental noise had significant effect on depressive symptoms. The greater the noise level in the living environment, the greater probability of experiencing mild-to-moderate depressive symptoms. These findings provide a basis for establishing noise-related public health policies and suggests the need for continued efforts to manage noise pollution, which has the potential to adversely affect mental well-being. Tailoring noise control strategies to the specific environmental conditions of each region could prevent health issues and mitigate the social burdens expected due to noise pollution.

### Supplementary Information

Supplementary Table 1.

## Data Availability

The datasets analyzed in the present study are publicly accessible. First, the 2019 Korean Community Health Survey (CHS) is available online: https://chs.kdca.go.kr/. Second, the 2019 Korean environmental noise measurement data is available online: https://www.noiseinfo.or.kr/noise/data.do/.
