# Supplementary material for: Depressive symptoms of people living in areas with high exposure to environmental noise: a multilevel analysis
Source: Sci Rep. 2024 Jun 24;14:14450. doi: 10.1038/s41598-024-65497-0 (PMC11196651; doi:10.1038/s41598-024-65497-0)
Supplement: Supplementary file 1 — Supplementary Table 1. [file 41598_2024_65497_MOESM1_ESM.docx]

| **Supplementary table. Results of subgroup analysis stratified by independent variables** | | | | | | | | | | | | | | |
| --- | --- | --- | --- | --- | --- | --- | --- | --- | --- | --- | --- | --- | --- | --- |
| **Variables** | | **Depressive symptoms** | | | | | | | | | | | | |
|  |  | **Environmental noise^a^** | | | | | | | | | | | | |
|  |  | **Q1 [lowest]** | **Q2** | | | | **Q3** | | | | **Q4 [highest]** | | | |
|  |  | **aOR** | **aOR** | **95% CI** | | | **aOR** | **95% CI** | | | **aOR** | **95% CI** | | |
| ***Individual level*** | |  |  |  |  |  |  |  |  |  |  |  |  |  |
| **Sex** |  |  |  |  |  |  |  |  |  |  |  |  |  |  |
|  | Men | 1.00 | 1.18 | (0.58 | - | 2.39) | 1.31 | (0.83 | - | 2.06) | 1.68 | (1.05 | - | 2.69) |
|  | Women | 1.00 | 1.30 | (0.72 | - | 2.37) | 1.38 | (0.94 | - | 2.02) | 1.48 | (1.00 | - | 2.21) |
| **Age** |  |  |  |  |  |  |  |  |  |  |  |  |  |  |
|  | ≤19 | 1.00 | 0.85 | (0.12 | - | 6.05) | 1.16 | (0.35 | - | 3.82) | 2.37 | (0.73 | - | 7.72) |
|  | 20~39 | 1.00 | 1.17 | (0.62 | - | 2.23) | 1.26 | (0.82 | - | 1.92) | 1.16 | (0.75 | - | 1.79) |
|  | 40~59 | 1.00 | 0.83 | (0.44 | - | 1.57) | 1.28 | (0.85 | - | 1.93) | 1.40 | (0.91 | - | 2.15) |
|  | 60+ | 1.00 | 1.74 | (0.87 | - | 3.50) | 1.42 | (0.91 | - | 2.22) | 1.80 | (1.13 | - | 2.87) |
| **Residential area** | |  |  |  |  |  |  |  |  |  |  |  |  |  |
|  | Seoul and metropolitan cities | 1.00 | 1.74 | (0.87 | - | 3.50) | 1.42 | (0.91 | - | 2.22) | 1.80 | (1.13 | - | 2.87) |
|  | Small cities and rural | 1.00 | 1.57 | (0.81 | - | 3.04) | 1.49 | (0.89 | - | 2.51) | 1.56 | (0.93 | - | 2.63) |
| **Education level** | |  |  |  |  |  |  |  |  |  |  |  |  |  |
|  | Low | 1.00 | 1.81 | (0.88 | - | 3.74) | 1.43 | (0.92 | - | 2.24) | 1.77 | (1.10 | - | 2.84) |
|  | Middle | 1.00 | 1.01 | (0.50 | - | 2.03) | 1.19 | (0.77 | - | 1.86) | 1.40 | (0.88 | - | 2.23) |
|  | High | 1.00 | 1.16 | (0.61 | - | 2.23) | 1.35 | (0.88 | - | 2.06) | 1.48 | (0.95 | - | 2.30) |
| **Occupation** | |  |  |  |  |  |  |  |  |  |  |  |  |  |
|  | White-collar | 1.00 | 1.25 | (0.60 | - | 2.60) | 1.33 | (0.81 | - | 2.18) | 1.25 | (0.74 | - | 2.10) |
|  | Blue-collar | 1.00 | 1.13 | (0.56 | - | 2.28) | 1.14 | (0.70 | - | 1.83) | 1.48 | (0.92 | - | 2.41) |
|  | Pink-collar | 1.00 | 1.26 | (0.65 | - | 2.46) | 1.67 | (1.06 | - | 2.64) | 1.55 | (0.95 | - | 2.52) |
|  | Else | 1.00 | 1.25 | (0.64 | - | 2.42) | 1.32 | (0.87 | - | 2.00) | 1.59 | (1.03 | - | 2.45) |
| **Household Income level^b^** | |  |  |  |  |  |  |  |  |  |  |  |  |  |
|  | Q1 | 1.00 | 1.27 | (0.67 | - | 2.42) | 1.41 | (0.93 | - | 2.14) | 1.41 | (0.93 | - | 2.14) |
|  | Q2 | 1.00 | 1.41 | (0.69 | - | 2.88) | 1.56 | (0.98 | - | 2.49) | 1.69 | (1.04 | - | 2.75) |
|  | Q3 | 1.00 | 0.98 | (0.47 | - | 2.07) | 1.56 | (0.96 | - | 2.55) | 1.72 | (1.04 | - | 2.84) |
|  | Q4 | 1.00 | 1.10 | (0.54 | - | 2.23) | 1.12 | (0.67 | - | 1.89) | 1.48 | (0.89 | - | 2.46) |
| **Housing type** | |  |  |  |  |  |  |  |  |  |  |  |  |  |
|  | Common house | 1.00 | 1.23 | (0.65 | - | 2.31) | 1.31 | (0.87 | - | 1.97) | 1.70 | (1.11 | - | 2.60) |
|  | Apartment | 1.00 | 1.28 | (0.69 | - | 2.36) | 1.38 | (0.94 | - | 2.04) | 1.37 | (0.91 | - | 2.07) |
| **Smoking** | |  |  |  |  |  |  |  |  |  |  |  |  |  |
|  | Yes | 1.00 | 0.80 | (0.41 | - | 1.56) | 1.34 | (0.87 | - | 2.07) | 1.79 | (1.15 | - | 2.78) |
|  | No | 1.00 | 1.35 | (0.73 | - | 2.48) | 1.34 | (0.91 | - | 1.98) | 1.48 | (0.99 | - | 2.23) |
| **Drinking** | |  |  |  |  |  |  |  |  |  |  |  |  |  |
|  | Yes | 1.00 | 1.05 | (0.58 | - | 1.90) | 1.28 | (0.87 | - | 1.88) | 1.39 | (0.93 | - | 2.08) |
|  | No | 1.00 | 1.62 | (0.78 | - | 3.38) | 1.43 | (0.90 | - | 2.28) | 1.72 | (1.06 | - | 2.81) |
| **Chronic diseases** | |  |  |  |  |  |  |  |  |  |  |  |  |  |
|  | Yes | 1.00 | 1.36 | (0.67 | - | 2.74) | 1.34 | (0.86 | - | 2.09) | 1.65 | (1.03 | - | 2.62) |
|  | No | 1.00 | 1.18 | (0.65 | - | 2.14) | 1.34 | (0.91 | - | 1.97) | 1.46 | (0.98 | - | 2.18) |
| **Subjective health status** | |  |  |  |  |  |  |  |  |  |  |  |  |  |
|  | Good | 1.00 | 1.12 | (0.51 | - | 2.44) | 1.50 | (0.90 | - | 2.48) | 1.66 | (0.99 | - | 2.79) |
|  | Bad | 1.00 | 1.26 | (0.70 | - | 2.28) | 1.31 | (0.90 | - | 1.91) | 1.50 | (1.01 | - | 2.22) |
| aOR, adjusted odds ratio, CI, confidence interval  ^a^ Environmental noise measurements were divided to quartiles (Q1: Leq≤66.08 dB; Q2: 66.08 dB<Leq≤68.75 dB; Q3: 68.75 dB<Leq≤70.53 dB; Q4: 70.53 dB<Leq).  ^b^ Household income level was categorized into quartiles (Q1: monthly income≤2 million won ($1,500); Q2: 2 million won ($1,500)<monthly income≤3.5 million won ($2,600); Q3: 3.5 million won ($2,600)<monthly income≤5 million won ($3,700); Q4: 5 million won($3,700) <monthly income). | | | | | | | | | | | | | | |
